# ‘Ray Pattern’ of Hand Joint Involvement

**DOI:** 10.31138/mjr.34.1.108

**Published:** 2023-03-31

**Authors:** J Sankar, Jithin Mathew, Sanjay Jain, Varun Dhir

**Affiliations:** Rheumatology Division, Department of Internal Medicine, Postgraduate Institute of Medical Education and Research, Chandigarh, India

**Keywords:** spondyloarthritis (SpA), ray pattern, HLA B27, enthesitis related arthritis (ERA), NSAIDS

**Figure 1.**
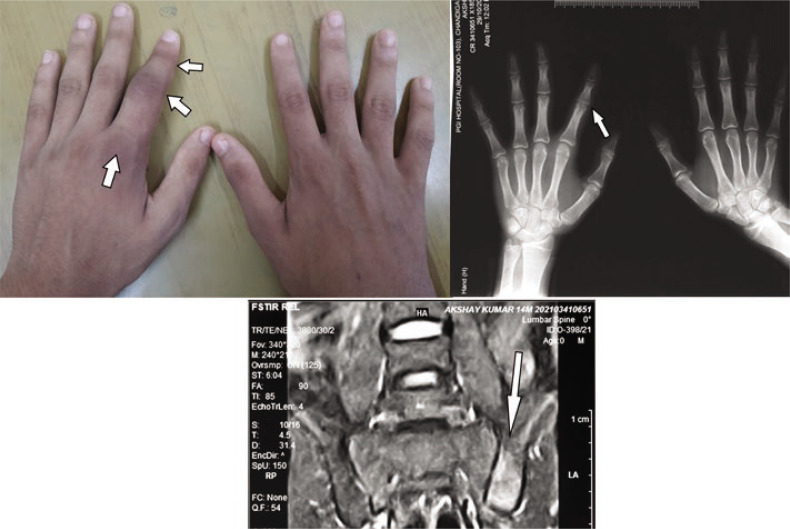
Upper panel (left): Swollen left second MCP, PIP and DIP joint resembling a ‘ray pattern’ of joint involvement. Upper panel (right): Hand radiograph showing reduced joint space of left second PIP joint with small erosions. Lower panel: MRI sacroiliac joint (STIR) showing bone marrow edema suggestive of acute sacroiliitis (left).

This 14-year-old boy presented with pain and swelling in the small joints of the left hand and pain in left buttock of 3-weeks duration. There was history of fever for 1-day preceding the onset of joint pain. On clinical examination, there was swelling and tenderness in the left second MCP, PIP, DIP in a ‘ray’ pattern (**Figure 1,** left upper panel) and there was left sacroiliac tenderness on the FABER test. Blood tests showed raised acute phase reactants (sedimentation rate 95mm/hr, C-reactive protein 58.7 mg/l) and a positive HLA B27 by polymerase chain reaction. Apart from these, other hematological, biochemical, urine, cultures, and viral markers were within normal limits. Radiograph of the chest and sonography of the abdomen were normal. Radiograph of the hands showed reduced joint space in the left second PIP joint with small erosions (**Figure 1**, right upper panel). Fluid sensitive images on MRI of sacroiliac joints showed bone marrow oedema in left sacroiliac joint (**Figure 1**, lower panel). A diagnosis of JIA- Enthesitis-related arthritis (ERA) was made and he was initiated on therapy with NSAIDS, low-dose prednisolone and sulfasalazine to which he responded.

A ‘ray’ pattern refers to involvement of all three joints of an affected digit, in contrast to the ‘row’ pattern where involvement of many similar joints occurs in a row, like many MCPs or PIPs (as in rheumatoid arthritis). The ray pattern has been used to distinguish psoriatic arthritis from rheumatoid arthritis both clinically and radiologically.^1,2^ However, as shown by this case of JIA-ERA, the ray pattern may be a generic feature of the peripheral involvement of the whole group of the spondyloarthritides (SpA). Besides, the dactylitis had also initially been thought to occur only in psoriatic arthritis, before it was recognised as a universal and defining SpA feature.^3^
